# EVALUATION OF ART ON THE BRAIN, A HEALTH APP FOR OLDER ADULTS, IN LONG-TERM CARE

**DOI:** 10.1093/geroni/igz038.2977

**Published:** 2019-11-08

**Authors:** Elizabeth P Howard, Tammy Retalic, Jessica Rogan

**Affiliations:** 1 CONNELL SCHOOL OF NURSING, BOSTON COLLEGE, CHESTNUT HILL, Massachusetts, United States; 2 HEBREW SENIORLIFE, BOSTON, Massachusetts, United States; 3 LIFE ENHANCEMENT, HEBREW SENIORLIFE, BOSTON, Massachusetts, United States

## Abstract

ArtOn the Brain is a mobile application that incorporates art history education, play and socialization for older adults. This pilot project examined its health and social impact among long term care residents. A convenience sample of 48 residents, 60+ years, with borderline intact to moderate cognitive impairment were recruited. Residents with mild or better cognitive status used the app individually on a tablet while the remaining participated in therapist-led group sessions. One-hour intervention occurred twice weekly. Primary outcomes, health and well-being, were measured with 5Q-5D-5L and Warwick-Edinburgh Mental Well-being tools at baseline and after 6 weeks. Secondary outcomes were mood, cognition, function and overall satisfaction with activity options and personal relationships. Pre-post paired t-test analyses of data from EQ-5D-5L and Warwick tools did not show a significant difference. There were no notable declines with cognitive performance, functional ability and depression post-intervention. Four activity options and three personal relationship items had statistically significant improvements post-intervention. Activity options addressed engagement in enjoyable activities on weekends and evenings, and opportunity to, spend time with other like-minded residents and explore new skills, interests. Personal relationships items included engagement in activities helpful to others and having people to do things with. ArtOn the Brain is a novel application suitable for long term care residents. While 6 week intervention did not show improvement in overall health and well-being, there were significant improvements with residents’ reported engagement in activity options and improvement of personal relationships. Longitudinal evaluations are needed to examine changes in health status and well-being.

